# CAR-T therapy for gastrointestinal cancers: current status, challenges, and future directions

**DOI:** 10.1590/1414-431X2024e13640

**Published:** 2024-10-14

**Authors:** Weidong Li, Yueming Huang, Xinhao Zhou, Bohao Cheng, Haitao Wang, Yao Wang

**Affiliations:** 1 Department of Gastrointestinal Surgery, Zhongshan City People's Hospital, Zhongshan, Guangdong, China

**Keywords:** CAR-T therapy, Gastrointestinal cancers, Target antigens

## Abstract

Chimeric antigen receptor (CAR) T-cell therapy is a revolutionary immunotherapeutic strategy that has shown efficacy in hematological malignancies. However, its application in solid tumors, particularly gastrointestinal cancers, faces significant challenges. These include the selection of target antigens, the complexity of the tumor microenvironment, and safety and toxicity concerns. This review provides a current overview of CAR-T therapy in various gastrointestinal cancers, such as esophageal, gastric, colorectal, pancreatic, and liver cancers. It discusses the limitations and future directions of CAR-T therapy in this context. This review highlights innovative strategies, including novel target antigens, multispecific CAR-T cells, armored CAR-T cells, and the development of universal CAR-T cells. These insights aim to inform ongoing research and foster advancements in CAR-T therapy for gastrointestinal cancers.

## Introduction

Gastrointestinal cancers are malignant tumors that arise in the organs of the digestive system, such as the esophagus, stomach, liver, pancreas, gallbladder, small intestine, and colorectum. Some of the risk factors for gastrointestinal cancers include chronic inflammation, infection, obesity, smoking, alcohol consumption, dietary habits, genetic mutations, and family history. Gastrointestinal cancers are among the most common and deadly cancers worldwide, accounting for about 25% of all cancer cases and 30% of all cancer deaths (1,2). Despite the advances in surgery, chemotherapy, radiotherapy, and targeted therapy, the prognosis of patients with gastrointestinal cancers remains poor, especially for those with advanced or metastatic disease (3). Therefore, there is an urgent need for novel and effective therapies that can improve the survival and quality of life of these patients. One of the most promising therapies for cancer is chimeric antigen receptor (CAR) T-cell therapy, which harnesses the power of the immune system to fight cancer cells. In this approach, patient T cells are genetically modified to express a CAR that converts T cells of any specificity into tumor-specific T cells. These cells can be expanded significantly and then readministered to the patient, so that they target and eliminate cancer cells, even in cases of extensive metastatic disease (4,5). CAR-T therapy has shown remarkable efficacy in hematological malignancies, leading to the approval of four CAR-T products by the Food and Drug Administration (FDA) for B cell malignancies (6).

However, the application of CAR-T therapy to solid tumors, especially gastrointestinal cancers, remains challenging due to several factors, such as the identification of suitable target antigens, the immunosuppressive tumor microenvironment, the potential toxicity and cost of CAR-T therapy, and the lack of standardized protocols and regulations (7). Despite these challenges, several clinical trials have explored the feasibility and safety of CAR-T therapy in gastrointestinal cancers, targeting antigens such as: CEA, HER2, MUC1, GPC3, and CLDN18.2 (8). Some of these trials have reported partial or complete responses in patients with advanced or refractory disease. However, the overall efficacy of CAR-T therapy in gastrointestinal cancers remains modest and variable (9).

This review aims to provide an update on the advances and challenges of CAR-T therapy in gastrointestinal cancers. We focus on the clinical application in peritoneal carcinomatosis from colorectal cancer and future prospects. We introduce the basic principles and mechanisms of CAR-T therapy, including its adverse effects. Then, we discuss the current status and results of CAR-T therapy in gastrointestinal cancers, emphasizing colorectal cancer and peritoneal carcinomatosis. Finally, we explore potential strategies to enhance the efficacy and safety of CAR-T therapy in gastrointestinal cancers, such as combination therapies, CAR design optimization, delivery, and the identification of biomarkers and predictors. This review systematically included both clinical and preclinical trials. We conducted a comprehensive literature search across multiple databases, including PubMed, Scopus, and Web of Science, to identify relevant current studies of our search. The inclusion criteria were designed to encompass studies that provided insights into the efficacy, safety, and mechanisms of CAR-T therapy in gastrointestinal cancers. For clinical studies, our inclusion criteria were as follows: studies that specifically evaluated the efficacy and safety of CAR-T therapy in gastrointestinal cancers, clinical trials that had reached at least Phase I to ensure a minimum level of clinical data, and preclinical studies that provided significant insights into the mechanisms of action or potential of CAR-T therapy in gastrointestinal settings. Our exclusion criteria for clinical studies were: early-stage preclinical trials without substantial evidence of progression to clinical application and clinical trials that did not provide clear data on therapeutic outcomes or safety profiles. We also incorporated findings from both *in vitro* and *in vivo* assays. The *in vitro* studies provided valuable data on the specificity and cytotoxicity of CAR-T cells against cancer cell lines. *In vivo* studies, primarily using murine models, were included to assess the therapeutic potential and systemic effects of CAR-T therapy in a living organism, which are critical for understanding the translational impact of the preclinical findings.

## Construction and delivery of chimeric antigen receptors (CARs) to T cells: components and functions

CARs are modular synthetic receptors that consist of four main components: 1) an extracellular target antigen-binding domain; 2) a hinge region; 3) a transmembrane domain; and 4) one or more intracellular signaling domains. The target antigen-binding domain is usually derived from a monoclonal antibody or a single-chain variable fragment (scFv) that recognizes a specific antigen on cancer cells (10,11). The hinge region provides flexibility and stability to the CAR and affects its conformation and function. The transmembrane domain anchors the CAR to the T cell membrane and can also influence the signaling and clustering of the CAR (12). The intracellular signaling domains are responsible for activating the T cell upon antigen recognition and can modulate the proliferation, survival, differentiation, and effector functions of the CAR-T cells.

The design and generation of CARs have evolved over time, resulting in different generations of CARs with different signaling domains. The first-generation CARs had only one intracellular signaling domain, usually derived from the CD3ζ chain of the T cell receptor (TCR) complex. The second-generation CARs added a co-stimulatory domain, such as CD28 or 4-1BB, to enhance the activation and persistence of CAR-T cells. The third-generation CARs incorporated two or more co-stimulatory domains, such as CD28, 4-1BB, OX40, or CD27, to further improve the function and durability of CAR-T cells. The fourth-generation CARs, also known as TRUCKs (T cells redirected for universal cytokine-mediated killing), incorporated an inducible cytokine gene, such as interleukin (IL)-12 or IL-15, to modulate the tumor microenvironment and recruit other immune cells (11,13).

**Figure 1:**
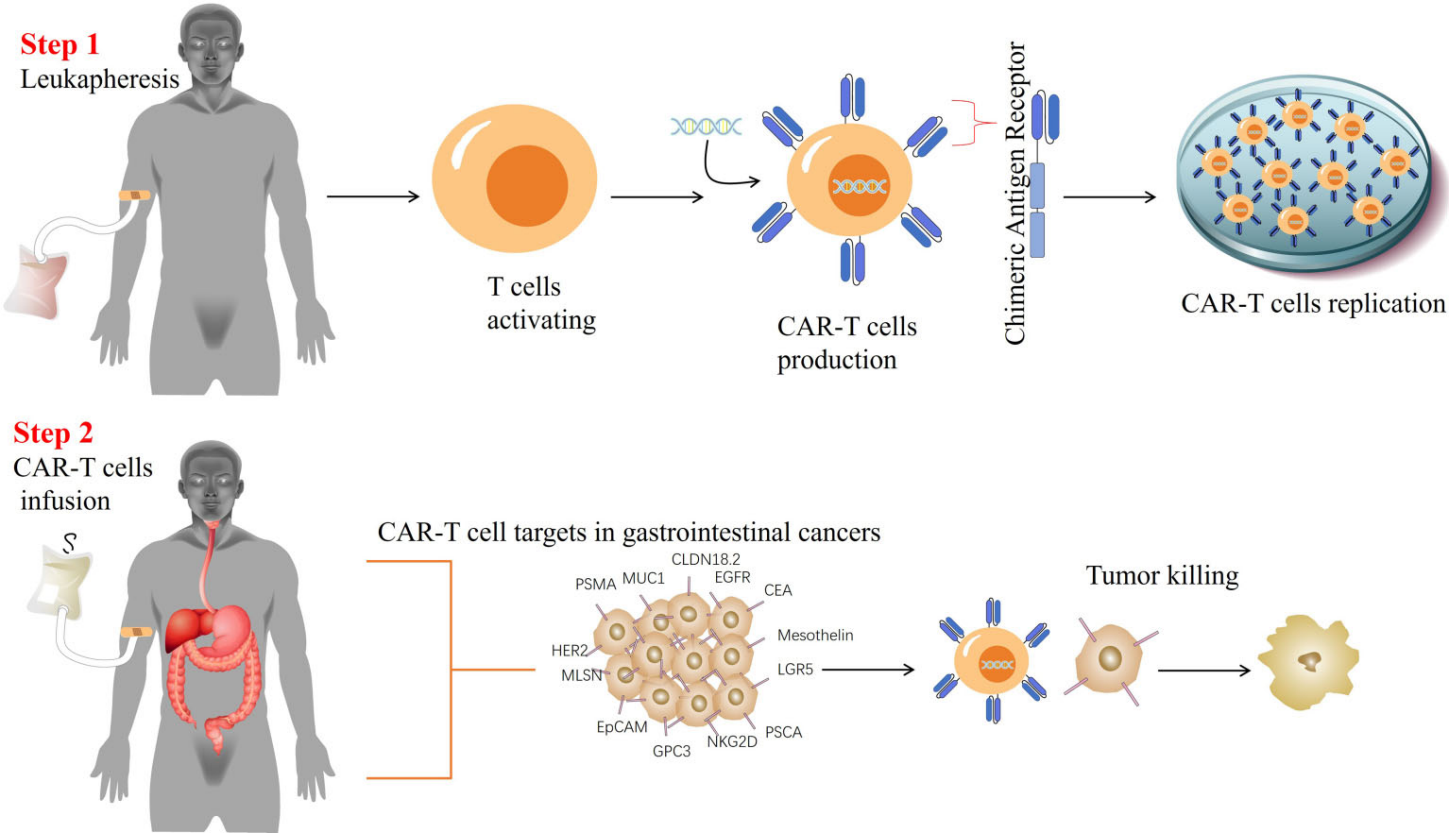
Chimeric antigen receptor (CAR)-T cell therapy utilized in the battle against gastrointestinal cancers. Initially, human T cells are harvested, carefully chosen, and activated in a controlled environment. Genetic modification is then conducted to prompt CAR expression within the T cells. The resulting CAR-T cells undergo expansion and formulation, adhering to stringent quality control measures to produce the final CAR-T cell product. This product is subsequently administered to the patient, with the anticipated outcome of targeting various gastrointestinal cancers antigens to eliminate tumor cells.

The construction and delivery of CARs to T cells involve several steps (Figure 1): 1) isolation of T cells from the patient's blood by leukapheresis; 2) transduction of T cells with a viral or non-viral vector carrying the CAR gene; 3) expansion and activation of the CAR-T cells *in vitro*; and 4) infusion of the CAR-T cells back to the patient (14). The most commonly used viral vectors for transducing T cells are retroviruses or lentiviruses, which integrate the CAR gene into the host genome and ensure stable expression. However, viral vectors have some drawbacks, such as high cost, potential immunogenicity, insertional mutagenesis, and limited cargo capacity (15). Non-viral vectors, such as plasmids or transposons, are cheaper, safer, and more versatile alternatives that can deliver larger or multiple genes to T cells. However, non-viral vectors have lower transduction efficiency and transient expression compared to viral vectors (16).

## Current status of CAR-T therapy in gastrointestinal cancers

CAR-T therapy has shown remarkable efficacy in hematological malignancies, but its application in solid tumors, such as gastrointestinal cancers, remains challenging. Currently, CAR-T therapy has been explored in various types of gastrointestinal cancers, such as esophageal cancer, gastric cancer, colorectal cancer, pancreatic cancer, and liver cancer. One of the challenges in CAR-T therapy for gastrointestinal cancers is identifying a suitable target antigen that is selectively expressed by tumor cells and not by normal cells. Here, we summarize some of the recent studies of CAR-T therapy targeting various antigens in different types of gastrointestinal cancers.

### Esophageal cancer and gastric cancer

Esophageal and gastric cancers are malignancies affecting the upper gastrointestinal tract. There are two primary histological subtypes of esophageal cancer, namely squamous cell carcinoma and adenocarcinoma, whereas gastric cancer is predominantly adenocarcinoma. Both esophageal cancer and gastric cancer are more prevalent in males than females, and are frequently linked to smoking, alcohol consumption, acid reflux, *Helicobacter pylori* infection, as well as genetic factors (7).

One potential therapeutic target for esophageal and gastric cancers is human epidermal growth factor receptor 2 (HER2), which exhibits overexpression in certain cases of these malignancies. A phase I clinical trial (NCT02713984) conducted by Southwest Hospital in China is currently evaluating the safety and efficacy of HER2-targeting CAR-T cells in patients with HER2-positive advanced solid tumors, including esophageal cancer and gastric cancer (9). This study aims to confirm the adverse effects of CAR-T cell therapy in gastric cancer patients, including cytokine storm response and other potential complications. Additionally, the persistence of CAR-T cells, tumor elimination efficacy, and disease status post-treatment will be evaluated. A recent study by Budi et al. (17) reported the development of an effective HER2-targeting CAR-T cell that showed promising anti-tumor activity against HER2-positive esophageal cancer cells *in vitro* and *in vivo*. Another preclinical study by Yang et al. reported the development of a novel HER2-targeting CAR-T cell that incorporated a CD28 costimulatory domain and a PD-1 blocking domain to enhance its function and persistence against HER2-positive gastric cancer cells (18). In clinical practice, monoclonal antibodies are commonly employed as a therapeutic approach. One example is the anti-HER2 monoclonal antibody trastuzumab, which has significantly enhanced the survival rates of patients with advanced gastric and esophageal cancers (19). The development of anti-HER2 CAR-T cells, despite the higher production costs compared to monoclonal antibodies like trastuzumab, is driven by several factors that highlight the potential advantages of CAR-T therapy. Firstly, CAR-T cells can provide long-lasting surveillance against tumor recurrence due to their ability to persist in the body as memory cells, potentially leading to longer remission periods compared to the transient effects of monoclonal antibodies. Secondly, CAR-T cells have the potential to penetrate solid tumors more effectively than monoclonal antibodies, which can be hindered by the dense extracellular matrix of tumors. In addition, tumors may develop resistance to monoclonal antibodies through various mechanisms, such as downregulation of the target antigen. CAR-T cells can be engineered to recognize multiple antigens, reducing the likelihood of tumor escape (20).

Cluster of differentiation 276 (CD276), which is a transmembrane protein that is involved in immune regulation and tumor progression is highly expressed in esophageal cancer tissues and cell lines, but not in normal tissues. In 2019, a phase I clinical trial (NCT04025216) was underway to evaluate the safety and efficacy of CD276-targeting CAR-T cells in patients with advanced solid tumors, including esophageal cancer. However, more recently, the sponsor deemed the risk/benefit analysis unfavorable and subsequently terminated the study (21). A recent study by Xuan et al. (22) reported the development of a CD276-targeting CAR-T cell that induced regression of esophageal cancer xenografts in mice and showed superior cytotoxicity and cytokine production than CD19-targeting CAR-T cells. This study tested the anti-tumor efficacy of CD276.CAR-T cells *in vivo* using subcutaneous and orthotopic xenograft mouse models of ESCC. The results showed that intratumoral injection of CD276.CAR-T cells induced regression of established CD276-positive tumors and prolonged mouse survival, while CD19-targeting CAR-T cells had no effect.

Epidermal growth factor receptor (EGFR) is a transmembrane protein that is involved in cell proliferation and survival. EGFR is overexpressed in about 50% of esophageal cancers and 30% of gastric cancers and is associated with poor prognosis and resistance to chemotherapy. A recent study by Cheng et al. (10) showed that the EGFR-targeting CAR-T cells exhibit significant anti-tumor activity against EGFR-positive esophageal cancer cells *in vitro* and *in vivo*. The results showed that EGFR.CAR-T cells selectively killed EGFR-positive cells and produced high levels of interferon-γ (IFN-γ), tumor necrosis factor-α (TNF-α), IL-2, and IL-6. In addition, intratumoral injection of EGFR.CAR-T cells significantly suppressed the growth of established EGFR-positive tumors, while CD19-targeting CAR-T cells had no effect. A phase I open-label, non-randomized study (NCT03618381) is currently evaluating the safety and efficacy of EGFR-targeting CAR-T cells in patients with advanced solid tumors, including esophageal cancer and gastric cancer in children and young adults at Seattle Children's Hospital. Recently, several trials have compared the efficacy of immunotherapy using monoclonal antibodies against EGFR. The results of these trials may provide further valuable insights into tumor biology and the interaction between the immune system and cancer cells (23,24). For instance, the efficacy and safety data from these trials can inform the design of CAR constructs, the selection of co-stimulatory domains, and the identification of potential toxicities associated with targeting EGFR. Additionally, understanding the mechanisms of resistance to EGFR-targeted monoclonal antibodies can guide the development of CAR-T cells that are less susceptible to similar resistance pathways. This could involve targeting multiple epitopes of EGFR or combining CAR-T therapy with other treatments to prevent the emergence of resistant clones.

Carcinoembryonic antigen (CEA) is a glycoprotein that is overexpressed in esophageal and gastric cancer and other cancers. A phase I interventional clinical trial (NCT02349724) is currently evaluating the safety and efficacy of CEA-targeting CAR-T cells (CEA-TCB) in combination with atezolizumab, an anti-PD-L1 antibody, in patients with advanced CEA-positive solid tumors, including esophageal cancer and gastric cancer. CEA-TCB is a bispecific antibody that simultaneously targets CEA on tumor cells and CD3 on T cells, thereby facilitating their interaction and promoting T cell-mediated cytotoxicity. A study conducted by Chi et al. (25) aimed to investigate the potential of recombinant human IL-12 (rhIL-12) in augmenting the anti-tumor efficacy of CAR-T cells that target CEA in gastric cancer. This study found that rhIL-12 significantly increased the activation, proliferation, and cytotoxicity of CEA-targeting CAR-T cells *in vitro*, as well as their expansion and persistence *in vivo*. The combination of CEA-targeting CAR-T cells and rhIL-12 showed superior anti-tumor activity than CEA-targeting CAR-T cells alone in inhibiting the growth of gastric cancer xenografts in mice.

Mesothelin (MSLN) is a cell surface protein that is normally restricted to the mesothelial cells but is significantly overexpressed in esophageal and gastric cancer and others. MSLN expression has been correlated with poor prognosis and resistance to chemotherapy in esophageal cancer and gastric cancer patients. A recent study by Sotoudeh et al. (26) reported the development of a MSLN-targeting CAR-T cell that showed significant anti-tumor activity against MSLN-positive esophageal cancer cells *in vitro* and *in vivo*. A phase I clinical trial conducted by Chinese PLA General Hospital (NCT03747965) is currently evaluating the feasibility and safety of CRISPR-Cas9 mediated PD-1 gene-knockout CAR-T cells in patients with MSLN-positive multiple solid tumors, including esophageal cancer and gastric cancer.

Claudin-18.2 (CLDN18.2) is a tight junction protein that is normally expressed in the gastric epithelium but is aberrantly overexpressed in esophageal and gastric cancer and others. CLDN18.2 expression has been correlated with tumor invasion, metastasis, and poor survival in esophageal cancer and gastric cancer patients. A recent study by Chen et al. (8) reported the development of a CLDN18.2-targeting CAR-T cell that showed specific cytotoxicity against CLDN18.2-positive esophageal cancer cells *in vitro* and induced tumor regression *in vivo*. Additionally, a multicenter, open-label Phase 1 clinical trial (NCT05472857) was conducted at Changhai Hospital in China to assess the safety and efficacy of autologous claudin18.2 CAR-T cell therapy for advanced gastric cancer patients with positive CLDN18.2 expression.

Natural killer group 2 member D (NKG2D) is an activating receptor that recognizes stress-induced ligands on tumor cells and infected cells. NKG2D ligands are expressed in esophageal cancer cells and gastric cancer cells, but not in normal epithelial cells. NKG2D-mediated recognition can trigger anti-tumor immune responses by natural killer cells and T cells. A recent study by Tao et al. (27) found CAR-T cells exhibited potent cytotoxicity against NKG2D ligand-positive gastric cancer cells *in vitro*, with a higher killing efficiency than non-transduced T cells or CAR-T cells targeting an irrelevant antigen. In a mouse model of subcutaneous gastric cancer, the CAR-T cells significantly suppressed tumor growth compared with non-transduced T cells or control CAR-T cells. In addition, cisplatin was shown to upregulate NKG2D ligand expression in gastric cancer cells and enhance the susceptibility to CAR-T cell-mediated cytotoxicity. Thus far, no clinical studies have been conducted by the medical community on the NKG2D-targeting CAR-T cell therapy in relation to esophageal and gastric cancer. As a result, there is still much to be learned about how NKG2D-targeting CAR-T cell therapy could potentially benefit patients suffering from these types of cancers.

### Colorectal cancer

Colorectal cancer is a malignancy that affects the colon and rectum, ranking as the third most prevalent neoplasm and second leading cause of cancer-related mortality worldwide. This disease is frequently associated with aging, obesity, physical inactivity, tobacco use, alcohol consumption, dietary habits, family history, and genetic mutations (28).

One potential antigen target for colorectal cancer is leucine-rich repeat-containing G-protein-coupled receptor 5 (LGR5), a stem cell marker that exhibits high expression levels in colorectal cancer and other malignancies. The upregulation of LGR5 has been associated with unfavorable prognosis and resistance to chemotherapy among patients with colorectal cancer. Previous literature indicates that while LGR5 expression may decrease as colorectal cancer advances, it still plays a crucial role in the cancer stem cell population that contributes to tumor growth and metastasis (29). Moreover, LGR5-positive cells have been identified in metastatic lesions, suggesting that these cells retain their tumorigenic potential and could be viable targets for therapy. The rarity of early diagnosis in colorectal cancer presents a challenge for any targeted therapy. However, targeting LGR5 could still be considered a promising approach for several reasons. Firstly, LGR5 targeting could potentially eliminate cancer stem cells that are responsible for relapse and metastasis. Secondly, even with decreased expression in advanced stages, LGR5-positive cells may still be sufficiently present to be therapeutically relevant. Lastly, ongoing research and clinical trials are exploring the efficacy of targeting LGR5 in various stages of colorectal cancer, which could provide more definitive answers in the future (30,31). A phase I/IIa clinical trial (NCT05759728) is currently underway to assess the safety and efficacy of CNA3103, a novel LGR5-targeting CAR-T cell therapy, in patients with metastatic colorectal cancer who have failed prior lines of chemotherapy. CNA3103 incorporates a CD28 costimulatory domain and a PD-1 blocking domain to enhance its function and persistence against LGR5-positive colorectal cancer cells. In a recent study, potential strategies for targeting Lgr5+ colorectal cancer cells are discussed, including the use of CAR-T cell therapy. The authors suggest that engineering T cells to express chimeric antigen receptors (CARs) that recognize tumor-associated antigens is a promising approach for treating colorectal cancer (32). They propose that LGR5 may be an appropriate target for CAR-T cell therapy due to its high expression in colorectal cancer and crucial role in maintaining colorectal cancer stem cells (CSCs). In addition to LGR5, CD166 has also been identified as a potential target due to its role in maintaining the stemness and tumorigenicity of colorectal CSCs (33). These studies highlight the importance of addressing the CSC markers in the context of CAR-T cell therapies for colorectal cancer.

CEA is also overexpressed in colorectal cancer. Currently, a phase I clinical trial (NCT02349724) is underway to evaluate the safety and efficacy of CEA-targeting chimeric antigen receptor T cells (CEA-TCB) in combination with atezolizumab, an anti-PD-L1 antibody, for patients with advanced CEA-positive solid tumors, including colorectal cancer (6). CEA-TCB is a bispecific antibody that binds both to CEA in tumor cells and CD3 in T cells, thereby linking them together and activating T cell-mediated cytotoxicity. A recent study has reported on a preclinical investigation of a novel CEA-targeting CAR-T cell construct that incorporates both a CD28 costimulatory domain and a CD3ζ signaling domain (34). The authors have evaluated the antitumor activity and toxicity of this construct *in vitro* and *in vivo* using human CRC cell lines as well as xenograft mouse models. They demonstrated the efficient killing of CEA+ CRC cells *in vitro* by CEA-targeting CAR-T cells, which also suppressed tumor growth and prolonged survival *in vivo*. Furthermore, these CAR-T cells exhibited minimal damage to normal tissues expressing low levels of CEA, such as liver and colon.

EGFR is overexpressed in about 80% of colorectal cancers and is associated with poor prognosis and resistance to chemotherapy. A recent study has demonstrated the efficacy and safety of a novel EGFR-targeting CAR-T cell construct that incorporates both CD28 costimulatory domain and CD3ζ signaling domain. The antitumor activity and toxicity of this construct were evaluated *in vitro* using human CRC cell lines, as well as *in vivo* using xenograft mouse models (35). This study demonstrated the efficient killing of EGFR+ CRC cells *in vitro* by EGFR-targeting CAR-T cells, which also suppressed tumor growth and prolonged survival *in vivo*. Furthermore, they exhibited minimal damage to normal tissues expressing low levels of EGFR, such as skin and lung. A phase I clinical trial (NCT03618381) is currently evaluating the safety and efficacy of EGFR-targeting CAR-T cells in patients with advanced solid tumors, including colorectal cancer.

The expression of MSLN has been associated with unfavorable prognosis and resistance to chemotherapy in patients with colorectal cancer. A recent study explores the potential of CAR-T cells in mediating tumor cell death for treating solid tumors including colorectal cancer. The results show that using MSLN-based CAR-T cells can significantly inhibit the growth of human colorectal and gastric cancer cells in mice without apparent toxic side effects (36). A phase I clinical trial (NCT03747965) is currently evaluating the safety and efficacy of MSLN-targeting CAR-T cells in patients with advanced solid tumors, including colorectal cancer. Using CRISPR-Cas9 technology, the researchers utilized PD-1 knockout in CAR-T cells and combined them with paclitaxel and cyclophosphamide pretreatment to modulate the MSLN-positive tumor immune microenvironment in cancer patients.

The expression of NKG2D is significantly elevated in colorectal cancer, whereas it is expressed at lower levels in normal colon epithelial cells. The recognition of NKG2D can activate anti-tumor immune responses by natural killer cells and T cells. In a study, NKG2D CAR-T cells exhibited dose-dependent cytotoxicity against human colorectal cancer cells compared to untransduced T cells (34). In addition, IL-2 and IFN-γ secreted by these cells were significantly higher than those secreted by untransduced T cells. Two separate clinical studies have been conducted to assess the safety and clinical activity of autologous and allogeneic NKG2D-based CAR-T therapy in metastatic colorectal cancer (mCRC). The SHRINK study (NCT03310008) evaluated the autologous NKG2D CAR (CYAD-01) while the alloSHRINK study (NCT03692429) evaluated an allogeneic analog of CYAD-01 (CYAD-101). Both studies assessed the safety and clinical efficacy of multiple infusions of NKG2D CAR-T cells administered concurrently with standard FOLFOX chemotherapy through a 3+3 design, evaluating three dose levels (DL) of NKG2D CAR-T cells. The concurrent administration of FOLFOX aims at improving the likeliness of clinical responses in solid tumors by favoring infiltration into and overcoming the immunosuppressive tumor microenvironment (TME), improving engraftment of CAR-T cells due to the lymphodepletion induced by the chemotherapy, and likely increasing the NKG2D ligand expression in tumor tissues targeted by the NKG2D CARs (37).

HER2 also has emerged as a validated target for CAR-T therapy, with recent reports indicating its overexpression in colorectal cancer, thus presenting a potential therapeutic avenue for colorectal cancer treatment (38). In a recent study by Xu et al. (39), the researchers evaluated HER2 as a promising target for metastatic colorectal cancer (mCRC) therapy using flow cytometry and tissue microarray (TMA) analysis, along with a 9-year survival follow-up. HER2-specific CAR-T cells demonstrated potent cytotoxicity and cytokine secretion against CRC cells *in vitro*. Moreover, in mouse xenograft models, HER2 CAR-T cells exhibited significant inhibition of CRC progression across three different xenograft models. Notably, HER2 CAR-T cells displayed heightened efficacy against HER2-positive colorectal cancer in patient-derived tumor xenograft (PDX) models, showcasing potent immunotherapeutic potential for mCRC in metastatic xenograft mouse models. These findings underscore the promise of HER2 CAR-T cells as an emerging immunotherapy for the treatment of mCRC.

### Pancreatic cancer

Pancreatic cancer is a highly lethal malignancy, with a 5-year survival rate of less than 10%. It is anticipated to become the second leading cause of cancer-related mortality in the United States by 2030. Current treatment options are limited and ineffective, and there is an urgent need for novel therapeutic strategies. The pancreatic TME presents significant barriers to the infiltration and efficacy of CAR-T cells. The dense fibroblast population, the extracellular matrix, and the poorly formed vascular system can impede the access of CAR-T cells to tumor cells. However, recent studies have explored various strategies to enhance CAR-T cell infiltration, such as engineering CAR-T cells to overexpress chemokine receptors that match the chemokine profile of the pancreatic TME (40). Additionally, modifying the TME to reduce fibrosis and enhance vascularization has also been investigated to improve CAR-T cell access (41). Looking towards future perspectives, the development of CAR-macrophages (CAR-Ms) may also represent an exciting avenue in pancreatic tumor therapy. CAR-Ms can potentially overcome some of the limitations faced by CAR-T cells in solid tumors. Macrophages are naturally adept at infiltrating solid tumors and can be reprogrammed from a pro-tumoral (M2) to an anti-tumoral (M1) phenotype. This reprogramming can enhance phagocytosis and antigen presentation, thereby exerting direct and indirect anti-pancreatic tumor effects (42).

A recent study was conducted to investigate the therapeutic efficacy of CAR-T cell therapy targeting CEA in pancreatic adenocarcinoma. Their results showed that the CAR-T cells expressed high levels of the CAR on their surface and secreted high levels of IFN-γ and TNF-α upon stimulation with CEA-positive pancreatic cancer cell lines. The CAR-T cells also exhibited potent cytotoxicity against CEA-positive pancreatic cancer cells *in vitro*, with a higher killing efficiency than non-transduced T cells or CAR-T cells targeting an irrelevant antigen (43). Another study has assessed the therapeutic potential of CAR-T therapy designed to target CEA in pancreatic ductal adenocarcinoma (PDAC). Through a functional assay conducted on diverse PDAC cell lines, the study explored the correlation between CEA expression levels and the effectiveness of anti-CEA-CAR-T treatment. A notable association emerged, indicating a substantial correlation between CEA expression levels and the therapeutic impact. To further validate these findings, the researchers established orthotopic PDAC xenograft mouse models. Upon administering anti-CEA-CAR-T, a notable therapeutic effect was observed exclusively in the cell line characterized by high CEA expression (44). Several clinical trials have been conducted or are ongoing to evaluate the safety and efficacy of CEA-targeting CAR-T cell therapy in patients with advanced or metastatic esophageal cancer or gastric cancer. Currently, a single-arm, open-label, dose-escalating + dose-expansion clinical study (NCT05538195) was conducted to evaluate the safety and efficacy of CEA-targeted CAR-T cell preparations and preliminarily observe the study drug in CEA-positive advanced malignant tumors. This study plans to enroll 60 patients with CEA-positive metastatic or unresectable solid tumors, such as gastric cancer, colon cancer, rectal cancer, esophageal cancer, and pancreatic cancer, who have progressed after at least two lines of chemotherapy. The patients will receive a single infusion of autologous CEA-targeted CAR-T cells after lymphodepletion with cyclophosphamide and fludarabine. The dose of CAR-T cells will be escalated from 1×10^7^ to 3×10^8^ cells per patient in four cohorts. The study is sponsored by Chongqing Precision Biotech Co., Ltd. and is currently recruiting participants at He'nan Cancer Hospital, China.

Recently, a second-generation CAR targeting pancreatic adenocarcinoma was developed by Lee et al. (45), incorporating an anti-MSLN single-chain variable fragment (scFv) and CD28/CD3ζ signaling domains. Their study found that the CAR-T cells expressed high levels of CAR on their surface and secreted high levels of IFN-γ and TNF-α upon stimulation with MSLN-positive pancreatic cancer cell lines. The CAR-T cells also exhibited potent cytotoxicity against MSLN-positive pancreatic cancer cells *in vitro*, with a higher killing efficiency than non-transduced T cells or CAR-T cells targeting an irrelevant antigen. A Phase I/II study, conducted by Massachusetts General Hospital, is currently enrolling participants in the United States to assess the safety and efficacy of MSLN-targeted CAR-T-cell therapy for patients with metastatic pancreatic cancer (NCT03323944). The study plans to enroll 30 patients with MSLN-positive metastatic pancreatic adenocarcinoma who have progressed after at least one line of chemotherapy. The patients will receive a single infusion of autologous MSLN-targeted CAR-T cells after lymphodepletion with cyclophosphamide and fludarabine. The dose of CAR-T cells will be fixed at 3×10^7^ cells/kg. The primary endpoint of this study is safety, which will be evaluated by assessing the incidence and severity of adverse events as well as dose-limiting toxicities. Secondary endpoints include objective response rate, progression-free survival, overall survival, and pharmacokinetics/pharmacodynamics of CAR-T cells.

Mucin 1, also referred to as MUC1, is a highly significant member of the mucin family due to its heavily glycosylated and phosphorylated membrane-bound structure. This remarkable protein plays an essential role in various physiological processes such as cell signaling, adhesion, and differentiation. A study conducted by Zhai et al. (46) examined the antigen-specific antitumor activity of CAR-Vγ9Vδ2 T cells that targeted MUC1-Tn antigen, a cancer-associated glycoform of MUC1. Vγ9Vδ2 T cells were expanded from peripheral blood mononuclear cells obtained from healthy volunteers using zoledronic acid and interleukin-2. CAR-Vγ9Vδ2 T cells were generated by transfection of lentivirus encoding MUC1-Tn CAR. CAR-Vγ9Vδ2 T cells demonstrated antigen-specific cytotoxicity against various cancer cell lines, including pancreatic adenocarcinoma, with similar or stronger effects than CAR-αβ T cells. Nonetheless, CAR-Vγ9Vδ2 T cells exhibited a shorter persistence, which could be ameliorated by the addition of IL-2 to sustain the functionality of CAR-Vγ9Vδ2 T cells upon consecutive stimulation from tumor cells. A study conducted by the First Affiliated Hospital of Harbin Medical University aims to recruit 10 patients with metastatic or unresectable pancreatic cancer who have progressed after at least two lines of chemotherapy (NCT03267173). These patients will receive a single infusion of autologous CAR-T cells without prior lymphodepletion, and the dose of MUC1-targeted CAR-T cells will be increased from 3×10^7^ to 3×10^9^ cells per infusion in six cohorts. The primary endpoint of this study is safety, which will be evaluated by the incidence and severity of adverse events as well as dose-limiting toxicities. The secondary endpoint is efficacy, which will be assessed based on the objective response rate according to the Response Evaluation Criteria in Solid Tumors (RECIST).

The EPH receptor A2 (EphA2), a member of the Eph receptor family, is significantly overexpressed in pancreatic cancer tissues. In a recent study, dimeric compounds were synthesized based on the potent EphA2-targeting peptide (135H12) to bind and activate the receptor (47). The molecular modeling, biophysical assays, and cellular assays characterized the dimeric agents. This study revealed that the dimeric agents exhibited superior binding affinity and agonistic activity compared to their monomeric counterparts. In pancreatic cancer cell lines, the dimeric agents induced EphA2 phosphorylation, internalization, and degradation. Furthermore, these agents effectively reversed the pro-migratory effects of EphA2 *in vitro* as demonstrated by wound healing and transwell assays. Shanghai Unicar-Therapy Bio-medicine Technology Co., Ltd. (China) is currently conducting a Phase I trial, which is a single-center, single-arm, open-label study aimed at evaluating the safety and efficacy of CAR-T cell therapy targeting EphA2 expressed on various solid tumors, including pancreatic cancer (NCT05003895). This study aims to enroll 18 patients with advanced solid tumors, including pancreatic cancer, who exhibit positive expression of EphA2 and have experienced disease progression after at least one line of standard therapy. The patients will undergo lymphodepletion with cyclophosphamide and fludarabine prior to receiving a single infusion of autologous CAR-T cells targeting EphA2. The dose of CAR-T cells will be increased in three cohorts, ranging from 1×10^7^ to 3×10^8^ cells/m^2^. The primary endpoint for this study is safety, which will be evaluated by the incidence and severity of adverse events as well as dose-limiting toxicities. Secondary endpoints include objective response rate, progression-free survival, overall survival, and pharmacokinetics/pharmacodynamics of CAR-T cells.

### Liver cancer

Several types of cancer can form in the liver. Hepatocellular carcinoma (HCC), the most prevalent liver cancer, originates in the primary liver cell type known as hepatocytes. Less frequently encountered are alternative forms of liver cancer, including intrahepatic cholangiocarcinoma and hepatoblastoma. There are several challenges in applying CAR-T therapy to liver cancer, including poor vascularization of the tumor cells hindering T cell infiltration and expression of immunosuppressive proteins by the cancer cells impeding T cell recognition and attack. Nevertheless, several preclinical studies and early clinical trials have shown promising results for CAR-T therapy in treating liver cancer.

Glypican-3 (GPC3) is a glycosylphosphatidylinositol-anchored protein that exhibits overexpression in HCC and other solid tumors. GPC3 plays a crucial role in cell proliferation, migration, and invasion, making it an attractive target for CAR-T therapy. Preclinical studies have demonstrated the efficacy of GPC3-targeting CAR-T cells *in vitro* and *in vivo*, resulting in effective elimination of GPC3-positive HCC cells, tumor regression, and prolonged survival in mouse models of HCC. Several strategies have been developed to enhance the specificity, safety, and efficacy of GPC3-targeting CAR-T cells, such as using dual-targeting CARs, armored CARs, switchable CARs, or combination therapies. Clinical studies (NCT02395250 and NCT03146234) have reported the safety and feasibility of GPC3-targeting CAR-T cell therapy in patients with advanced HCC who have failed prior treatments (48). The most common adverse events are pyrexia, fatigue, and nausea, however, no severe cytokine release syndrome or neurotoxicity has been observed. The objective response rate ranges from 15 to 33%, with some patients achieving partial response or stable disease. The median progression-free survival and overall survival are 3.8 and 7.7 months, respectively. There exists a positive correlation between the expansion and persistence of CAR-T cells and clinical responses.

EpCAM (epithelial cell adhesion molecule) is a protein that is expressed on the surface of many cancer cells, including liver cancer cells. It can function as a biomarker of cancer stem cells (CSCs) or circulating tumor cells (CTCs), and also participate in cancer progression by interacting with other signaling pathways. A recent study utilizing a mouse model of orthotopic human HCC demonstrated that chimeric antigen receptor CAR-T cells targeting EpCAM effectively suppressed tumor growth and prolonged survival (49). Additionally, the CAR-T cells induced apoptosis and necrosis in tumor cells, reduced angiogenesis, and increased infiltration of natural killer (NK) cells and CD8+ T cells within the tumor microenvironment. According to ClinicalTrials.gov, there is currently one registered trial (NCT02729493) recruiting patients with relapsed or refractory liver cancer for infusion of their own T cells that have been genetically modified to express a CAR targeting EpCAM-positive tumor cells. The primary outcome of this trial is the objective response rate (ORR), and the secondary outcomes include progression-free survival (PFS), overall survival (OS), duration of response (DOR), safety, and tolerability. To date, no study results have been posted on ClinicalTrials.gov for this study.

In addition to the common HCC discussed above, a recent study has delved into intrahepatic cholangiocarcinoma and CAR-T therapy. This type of cancer develops in the intrahepatic bile ducts and is occasionally classified as a form of liver cancer. The research findings have demonstrated that CAR-T cells targeting MUC1 possess a significant ability to hinder tumor growth and prolong survival in a mouse model of human intrahepatic cholangiocarcinoma. This effect is ascribed to the induction of apoptosis and necrosis in tumor cells, the upregulation of pro-inflammatory cytokines and chemokines, and the enhancement of CD4+ and CD8+ T cell infiltration within the tumor microenvironment (50).

## Current limitations and future perspectives of CAR-T therapy in gastrointestinal cancers

CAR-T therapy has demonstrated remarkable efficacy in hematological malignancies; nevertheless, its application in solid tumors, particularly gastrointestinal cancers, poses considerable challenges. As previously discussed, CAR-T therapy has been investigated across a spectrum of gastrointestinal cancers, including esophageal cancer, gastric cancer, colorectal cancer, pancreatic cancer, and liver cancer. However, the clinical experience and evidence of CAR-T therapy in gastrointestinal cancers are still limited and mostly based on phase 1 or phase 2 trials with small sample sizes and short follow-up periods (Table 1). Several limitations impede the clinical success of CAR-T therapy in the context of gastrointestinal cancers.

**Table 1:** Clinical trials of chimeric antigen receptor (CAR)-T cells in patients with gastrointestinal cancers.

Type of gastrointestinal cancer	Identifier	Study name	Status	Phase	Intervention	Location
Esophageal and gastric cancers	NCT02713984	A Clinical Research of CAR T Cells Targeting HER2 Positive Cancer	Recruiting	1	HER-2-targeting CAR-T cells infusion in HER2 positive cancers	China
Esophageal cancer	NCT04025216	A Study of CART-TnMUC1 in Patients with TnMUC1-Positive Advanced Cancers	Terminated	1	CART-TnMUC1 and chemotherapy	United States
Esophageal and gastric cancers	NCT03618381	EGFR806 CAR T Cell Immunotherapy for Recurrent/Refractory Solid Tumors in Children and Young Adults	Recruiting	1	Second generation 4-1BBζ EGFR806-EGFRt	United States
Gastric cancer and pancreatic cancer	NCT05472857	Clinical Study of CLDN18.2-targeting CAR T Cells in Advanced Solid Tumors with Positive CLDN18.2 Expression	Recruiting	1/2	Claudin 18.2 CAR-T	China
Gastric cancer	NCT02349724	A Clinical Research of CAR T Cells Targeting CEA Positive Cancer	Recruiting	1	Anti-CEA-CAR-T	China
Colorectal cancer	NCT03747965	Study of PD-1 Gene-knockout Mesothelin-directed CAR-T Cells with the Conditioning of PC in Mesothelin Positive Multiple Solid Tumors	Recruiting	1	Anti-Mesothelin-CAR T	China
Colorectal cancer	NCT05759728	A Study of CNA3103 (LGR5-targeted, Autologous CAR-T Cells) Administered to Subjects with Metastatic Colorectal Cancer	Recruiting	1/2	LGR5-targeted, Autologous CAR-T	Australia
Colorectal cancer	NCT03310008	Dose Escalation and Dose Expansion Phase I Study to Assess the Safety and Clinical Activity of Multiple Doses of NKR-2 Administered Concurrently with FOLFOX in Colorectal Cancer with Potentially Resectable Liver Metastases (SHRINK)	Recruiting	1	NKG2D CAR-T	Belgium
Colorectal Cancer	NCT03692429	alloSHRINK - Standard Chemotherapy Regimen and Immunotherapy with Allogeneic NKG2D-based CYAD-101 Chimeric Antigen Receptor T-cells	Recruiting	1	NKG2D-based CYAD-101 Chimeric Antigen Receptor T-cells	United States
Colorectal; gastric; and pancreatic cancer	NCT05538195	Evaluate the Safety and Efficacy of CEA-targeted CAR-T for CEA-positive Advanced Malignant Solid Tumors	Recruiting	1/2	CEA-targeted CAR-T cells	China
Pancreatic cancer	NCT03267173	Evaluate the Safety and Efficacy of CAR-T in the Treatment of Pancreatic Cancer	Recruiting	1	Mesothelin/PSCA/CEA/HER2/MUC1 CAR-T cells	China
Pancreatic cancer	NCT03323944	Phase I Study of Human Chimeric Antigen Receptor Modified T Cells (CAR T Cells) in Patients with Pancreatic Cancer	Recruiting	1	huCART-meso cells	United States
Hepatocellular carcinoma	NCT05003895	GPC3 Targeted CAR-T Cell Therapy in Advanced GPC3 Expressing Hepatocellular Carcinoma (HCC)	Recruiting	1	GPC3 Targeted CAR-T	United States
Hepatocellular carcinoma	NCT02395250	Anti-GPC3 CAR T for Treating Patients with Advanced HCC	Completed	1	anti-GPC3 CAR-T	China
Hepatocellular carcinoma	NCT03146234	CAR-GPC3 T Cells in Patients with Refractory Hepatocellular Carcinoma	Completed	1	anti-GPC3 CAR T	China
Liver neoplasms	NCT02729493	Study Evaluating the Efficacy and Safety with CAR-T for Liver Cancer Patients	Recruiting	2	EpCAM-targeting CAR-T cell therapy	China
Hepatocellular carcinoma and pancreatic cancer	NCT02587689	Phase I/II Study of Anti-Mucin1 (MUC1) CAR T Cells for Patients with MUC1+ Advanced Refractory Solid Tumor	Recruiting	1	MUC1-targeting CAR-T cell therapy	China

In CAR-T therapy for gastrointestinal cancers, a significant challenge lies in identifying a target antigen that is selectively expressed by tumor cells and not by normal cells. Many of the antigens targeted by CAR-T therapy in gastrointestinal cancers, including HER2, CEA, MUC1, and EGFR, are also present in normal tissues, potentially resulting in on-target off-tumor toxicity. Additionally, certain antigens may exhibit heterogeneity or downregulation by tumor cells, leading to antigen escape or resistance (51). Consequently, the discovery and validation of more specific and universally applicable antigens are imperative for advancing CAR-T therapy in the treatment of gastrointestinal cancers. Another challenge in CAR-T therapy for gastrointestinal cancers is the hostile tumor microenvironment, which may impair the infiltration, activation, proliferation, and persistence of CAR-T cells (52). The TME in gastrointestinal cancers is characterized by hypoxia, acidity, immunosuppression, and fibrosis, which may affect the function and survival of CAR-T cells. Moreover, the TME may also induce the expression of inhibitory molecules, such as PD-L1, on tumor cells, which may inhibit the cytotoxicity of CAR-T cells. Therefore, strategies to overcome the tumor microenvironment, such as combination therapy, preconditioning, and genetic engineering, need to be developed and optimized for CAR-T therapy in gastrointestinal cancers. Another major concern in CAR-T therapy for gastrointestinal cancers is the safety and toxicity, which may limit the dose and efficacy of CAR-T cells. The most common and severe adverse events associated with CAR-T therapy are cytokine release syndrome (CRS) and neurotoxicity, which may be life-threatening in some cases. Other potential adverse events include anaphylaxis, infection, graft-*versus*-host disease (GVHD), and insertional mutagenesis (53). Therefore, methods to monitor and manage the safety and toxicity of CAR-T therapy, such as biomarkers, grading systems, and antidotes, need to be established and implemented for CAR-T therapy in gastrointestinal cancers.

Despite these limitations, CAR-T therapy remains highly promising for treating gastrointestinal cancers. Numerous strategies and innovations aim to enhance the efficacy and safety of this therapy in this context. One such approach involves the identification and exploration of novel target antigens, such as GPC3, CLDN18.2, and MSLN, which exhibit higher specificity and lower toxicity compared to conventional antigens. Moreover, some of these novel antigens may be shared across various gastrointestinal cancers, paving the way for the development of a universal CAR-T therapy for this category of cancers.

Another avenue of exploration involves multispecific CAR-T cells, engineered to express two or more CARs targeting different antigens on tumor cells. This approach aims to improve efficacy and overcome resistance in gastrointestinal cancers. For instance, a dual-targeted CAR-T cell recognizing both HER2 and MUC1 has demonstrated superior antitumor activity and reduced antigen escape in gastric cancer models compared to single-targeted CAR-T cells.

Furthermore, the development of armored CAR-T cells is underway, where cells are modified to secrete cytokines, chemokines, or antibodies, thereby enhancing their function and survival in the TME. For example, an IL-12-secreting CAR-T cell targeting CEA has exhibited improved infiltration and persistence in colorectal cancer models.

The concept of universal CAR-T cells is also gaining momentum. These cells are designed to recognize a synthetic antigen expressed by tumor cells after the administration of a bi-specific antibody, potentially reducing toxicity and increasing therapy flexibility in gastrointestinal cancers. A notable example is a universal CAR-T cell recognizing FITC, demonstrating potent antitumor activity and minimal toxicity in various solid tumor models following the injection of a FITC-conjugated antibody.

In conclusion, CAR-T therapy is a promising approach for treating various types of gastrointestinal cancers, but it also faces some limitations and challenges, especially in solid tumors. More research and development are needed to overcome these challenges and improve the efficacy and safety of CAR-T therapy in gastrointestinal cancers. Future perspectives include the discovery and validation of novel target antigens, the engineering and optimization of multispecific, armored, and universal CAR-T cells, and the establishment and implementation of methods to monitor and manage the safety and toxicity of CAR-T therapy in gastrointestinal cancers.
